# Clinical Utility of Multigene Profiling Assays in Early-Stage Invasive Breast Cancer: An Ontario Health (Cancer Care Ontario) Clinical Practice Guideline

**DOI:** 10.3390/curroncol29040213

**Published:** 2022-04-09

**Authors:** Phillip Blanchette, Duvaraga Sivajohanathan, John Bartlett, Andrea Eisen, Harriet Feilotter, Rossanna Pezo, Gulisa Turashvili, Phillip Williams

**Affiliations:** 1 London Regional Cancer Program, Division of Medical Oncology, London Health Sciences Centre, London, ON N6A 4W9, Canada; 2 Department of Oncology, McMaster University, Hamilton, ON L8S 4L8, Canada; 3 Program in Evidence-Based Care, Cancer Care Ontario, Ontario Health, Hamilton, ON L8S 4L8, Canada; 4 Cancer Research UK Edinburgh Centre, Institute of Genetics and Cancer, The University of Edinburgh, Edinburgh EH4 2XR, UK; john.bartlett@ed.ac.uk; 5 Ontario Institute for Cancer Research, Toronto, ON M5G 0A3, Canada; 6 Department of Medical Oncology, Odette Cancer Centre, Sunnybrook Health Sciences Centre, Toronto, ON M4N 3M5, Canada; andrea.eisen@sunnybrook.ca (A.E.); rossanna.pezo@sunnybrook.ca (R.P.); 7 Department of Pathology and Molecular Medicine, Queen’s University, Kingston, ON K7L 2V7, Canada; hf4@queensu.ca; 8 Laboratory Genetics, Kingston Health Sciences Centre, Kingston, ON K7L 2V7, Canada; 9 Department of Pathology and Laboratory Medicine, Emory University Hospital, Atlanta, GA 30322, USA; gulisa.turashvili@emory.edu; 10 Department of Laboratory Medicine & Pathobiology, Sinai Health, Toronto, ON M5G 1X5, Canada; phillip.williams@sinaihealth.ca

**Keywords:** breast cancer, cancer guideline, multigene profiling assays, Oncotype DX, Mammaprint, Prosigna, EndoPredict, Breast Cancer Index, assay

## Abstract

Objective: The purpose of this guideline is to determine the clinical utility of multigene profiling assays in individuals with early-stage invasive breast cancer. Methods: This guideline was developed by Ontario Health (Cancer Care Ontario)’s Program in Evidence-Based Care (PEBC) through a systematic review of relevant literature, patient- and caregiver-specific consultation and internal and external reviews. Recommendation 1: In patients with early-stage estrogen receptor (ER)-positive/human epidermal growth factor 2 (HER2)-negative breast cancer, clinicians should consider using multigene profiling assays (i.e., Oncotype DX, MammaPrint, Prosigna, EndoPredict, and the Breast Cancer Index) to help guide the use of systemic therapy. Recommendation 2: In patients with early-stage node-negative ER-positive/HER2-negative disease, clinicians may use a low-risk result from Oncotype DX, MammaPrint, Prosigna, EndoPredict/EPclin, or Breast Cancer Index assays to support a decision not to use adjuvant chemotherapy. Recommendation 3: In patients with node-negative ER-positive/HER2-negative disease, clinicians may use a high-risk result from Oncotype DX to support a decision to offer chemotherapy. A high Oncotype DX recurrence score is capable of predicting adjuvant chemotherapy benefit. Recommendation 4: In postmenopausal patients with ER-positive/HER2-negative tumours and one to three nodes involved (N1a disease), clinicians may withhold chemotherapy based on a low-risk Oncotype DX or MammaPrint score if the decision is supported by other clinical, pathological, or patient-related factors. Recommendation 5: The evidence to support the use of molecular profiling to select the duration of endocrine therapy is evolving. In patients with ER-positive disease, clinicians may consider using a Breast Cancer Index (H/I) high assay result to support a decision to extend adjuvant endocrine therapy if the decision is supported by other clinical, pathological, or patient-related factors.

## 1. Introduction

Breast cancer is a common disease in Canada, with approximately 25,000 new cases per year [1]. Survival outcomes with early-stage breast cancer have significantly improved over time with advances in systemic therapy, especially adjuvant chemotherapy and endocrine therapy [2].

Breast cancer is a heterogenous disease classified traditionally by expression of the ER, PR and/or HER receptor. Clinical decision making regarding adjuvant systemic therapy may vary and is commonly influenced by patient, clinical and pathologic factors, including tumor size, histologic grade, lymph node status and ER, PR and HER2 expression, all of which have been shown to significantly influence the risk of disease recurrence [3]. Given the potential side effects and toxicity of systemic therapy, several molecular gene expression profiling tests have been developed to assess the risk of recurrence. The use of these assays is meant to improve clinical decision making and optimize the use of systemic therapy for breast cancer.

Clinical decision-making regarding the use of adjuvant chemotherapy has historically been based on a variety of factors, including breast cancer stage, tumor biology and patient characteristics, all of which can be used to target patients at higher risk of disease recurrence. However, treatment decision remains challenging, especially among ER-positive, HER2-negative invasive breast cancers that are often less responsive to chemotherapy and may derive more clinical benefit from endocrine therapy alone. Previous treatment recommendations were generated from population-based or clinical trial data and were not necessarily indicative of clinical benefit at an individual patient level [4,5]. This imprecision has resulted in the overuse of adjuvant chemotherapy in some breast cancer patients, with unnecessary exposure to side effects and potential toxicity [6]. To mitigate this, several molecular profiling tests have been developed and validated that classify tumours into low-, intermediate-, or high-risk categories for risk of disease recurrence. These multigene profiling assays are generally prognostic of breast cancer outcomes. Some may also predict the potential benefit from systemic therapy in terms of distant recurrence, IDFS, and OS [7]. Currently, several multigene profiling assays are approved by health regulatory agencies and supported for use by international breast cancer clinical guidelines. These assays are used in standard clinical practice to guide clinical decision making regarding the use of adjuvant chemotherapy for node-negative ER-positive/HER2-negative invasive breast cancer.

Ontario Health (Cancer Care Ontario)’s Program in Evidence-Based Care, together with the Molecular Oncology and Testing Advisory Committee, developed the present guideline reviewing several multigene expression assays, including Oncotype DX (Exact Sciences Corporation, Madison, WI, USA), Mammaprint (Agendia, Irvine, CA, USA), Prosigna (Veracyte, South San Francisco, CA, USA) EndoPredict (Myriad Genetics, Inc., Zurich, Switzerland), and Breast Cancer Index (Biotheranostics, Inc., San Diego, CA, USA).

Another assay, IHC4, was not included, given potential concerns regarding the reproducibility of the Ki67 measurement across pathology laboratories. IHC4 is not a commercially available test; however, it can be calculated on the basis of ER, PR, and HER2 expression and Ki67 scoring [8]. The Working Group decided not to focus its investigation on the utility of multigene profiling assays with regard to supporting clinical decision making for neoadjuvant chemotherapy or radiation therapy, given the number of ongoing trials.

The objectives of the current review were to assess the clinical utility of Oncotype DX, MammaPrint, Prosigna, EndoPredict, and Breast Cancer Index in terms of their ability to predict response to adjuvant chemotherapy and extended adjuvant endocrine therapy. A specific aim was to investigate the evidence for the use of these molecular profiling assays in the setting of either node-negative or node-positive ER-positive/HER2-negative breast cancer patients in guiding clinical decisions to withhold or offer adjuvant chemotherapy. Additionally, important patient factors impacting the utilization of molecular profiling results, including age at diagnosis and menopausal status, were of special interest in this review.

## 2. Methods

### 2.1. The Program in Evidence-Based Care (PEBC)

The PEBC is an initiative of the Ontario provincial cancer system, Ontario Health (Cancer Care Ontario). The PEBC produces evidence-based and evidence-informed guidance documents using the methods of the Practice Guidelines Development Cycle [9,10]. The process for the current guideline included a systematic review with interpretation of the evidence by the authors, who then drafted recommendations based on the evidence and expert consensus; internal review by content and methodology experts; and external review by clinicians and other stakeholders. The authors had expertise in medical oncology, pathology, medical genetics, and health research methodology.

Further details of the methods and findings of the systematic review that informed these recommendations have been published elsewhere [11]. Briefly, MEDLINE, EMBASE and the Cochrane Library were searched for studies that reported predictive data based on assay outcome (i.e., studies considering differential treatment effect). If there were no predictive studies available for either adjuvant chemotherapy or extended adjuvant endocrine therapy, then prognostic studies examining late recurrence (i.e., 5–10 years) were included. The risk of bias was assessed for each included RCT or retrospective analyses of RCTs, where the randomization was not broken using Cochrane’s Risk of Bias tool, http://handbook.cochrane.org/, accessed on 10 June 2021 (Part 2, Section 8.5). Criteria from the QUIPS tool were used to assess the risk of bias for all prognostic studies.

### 2.2. Patient and Caregiver-Specific Consultation Group

Patients/survivors/caregivers participated as Consultation Group members. They reviewed copies of the project plan, drafted recommendations and provided feedback on their comprehensibility, appropriateness, and feasibility to the Working Group’s Health Research Methodologist. The Health Research Methodologist relayed this feedback to the Working Group for consideration.

### 2.3. Internal Review

PEBC guidelines were reviewed by a panel of content experts (Expert Panel) and a methodology panel (Report Approval Panel). For the guideline document to be approved, 75% of the content experts who comprise the Expert Panel must cast a vote indicating whether or not they approve the document or abstain from voting for a specified reason, and of those that vote, 75% must approve the document. In addition, the PEBC Report Approval Panel, a three-person panel with methodology expertise, must unanimously approve the document.

### 2.4. External Review

Feedback on the approved draft guideline was obtained from content experts and the target users through two processes. Through the Targeted Peer Review, a small number of content experts were identified by the Guideline Development Group (GDG) and asked to review and provide feedback on the guideline document. Through Professional Consultation, which is intended to facilitate dissemination of the final guidelines to Ontario practitioners, relevant care providers and other potential users of the guideline were contacted and asked to provide feedback on the guideline recommendations through a brief online survey.

## 3. Results

The full systematic review provides more details of the methodologic characteristics and clinical outcomes [11].

### 3.1. Patient and Caregiver-Specific Consultation Group

Three patients/survivors/caregivers participated as Consultation Group members. The Consultation Group found the recommendations were clear and detailed, with adequate evidence to support each recommendation. It was also noted that the guideline addresses issues of concern to patients, such as treatment versus survival benefits, and takes into consideration the emotional impact of testing.

### 3.2. Internal Review

Three Report Approval Panel members, including the PEBC Scientific Director and two methodology experts, reviewed and approved the draft guideline in September 2021.

Of the 10 members of the Expert Panel, 8 members cast votes for an 80% response rate in August 2021. All of those who voted approved the document (100%).

### 3.3. External Review

After approval of the document at internal review, the authors circulated the draft document to external review participants for review and feedback. Four clinical experts from Ontario and British Columbia were identified by the Working Group to be targeted peer reviewers. Two agreed to be reviewers; one response was received. Table 1 summarizes the survey results.

For Professional Consultation, two hundred ninety-two individuals who practice in Ontario were contacted. Fifty-five (18.8%) responses were received. Thirty stated that they did not have interest in this area or were unavailable to review this guideline at the time. Table 2 summarizes the results of the survey responses from nine professionals.

## 4. Recommendations and Key Evidence

The target population for this guideline is individuals diagnosed with early-stage invasive breast cancer for whom further information is needed for prognosis and treatment decision making. In this guideline, early-stage invasive breast cancer is defined as stage I to III breast cancers that are surgically operable and do not have evidence of locally recurrent or distant metastatic disease with pT1-T3 or pN0-N1a based on surgical pathologic staging. The intended users of this guideline are clinicians and policymakers involved in the diagnosis and treatment of breast cancer.

**Figure 1:**
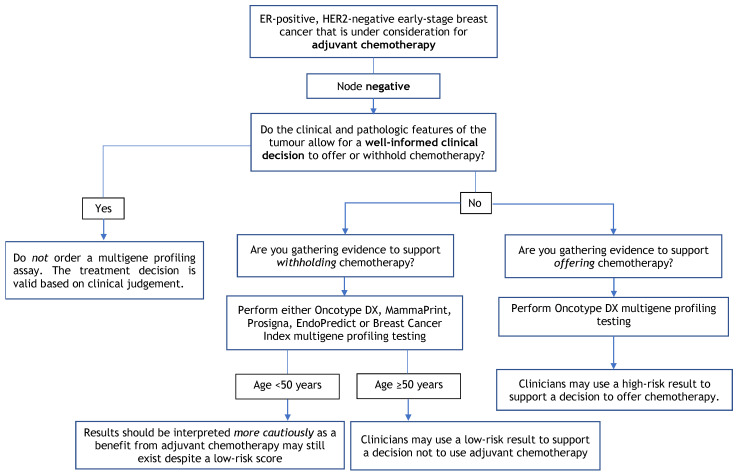
Multigene Profiling Assay Decision Tree for Adjuvant Chemotherapy in Node-Negative Patients.

**Figure 2:**
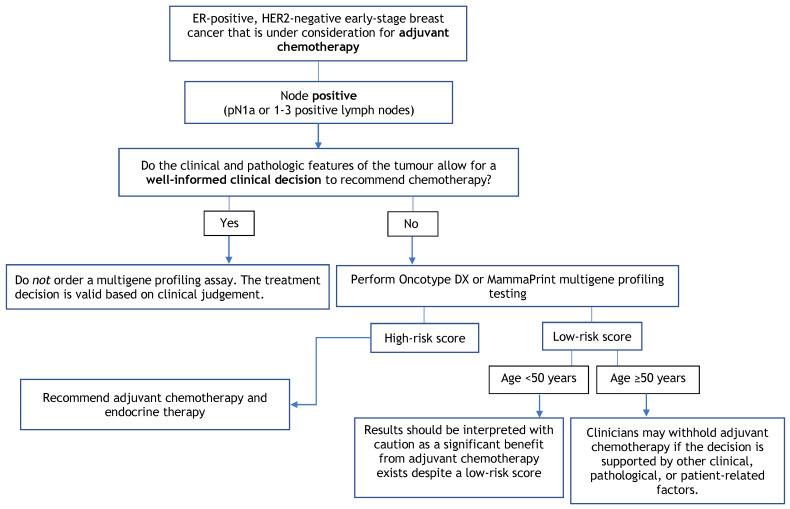
Multigene Profiling Assay Decision Tree for Adjuvant Chemotherapy in Node-Positive Patients.

**Figure 3:**
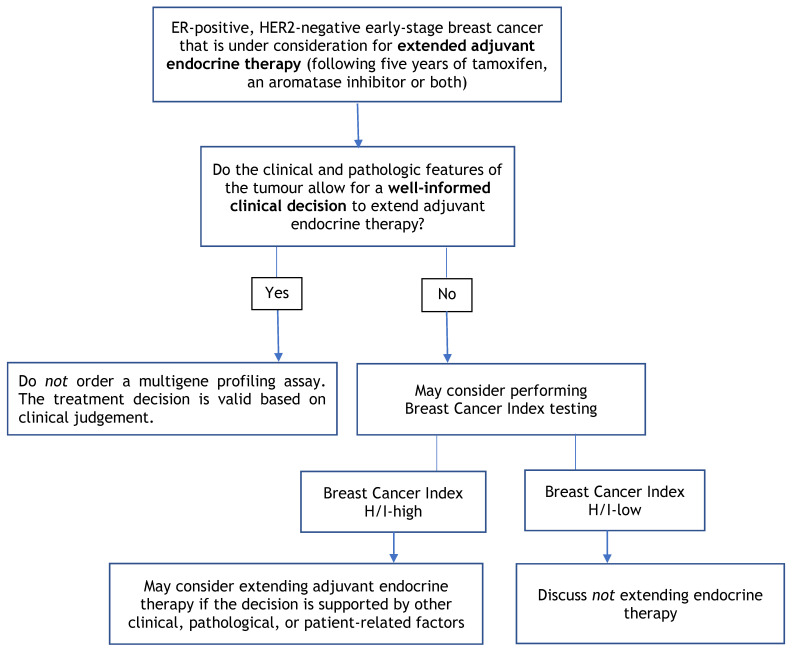
Multigene Profiling Assay Decision Tree for Extended Adjuvant Endocrine Therapy.

The purpose of this guideline is to determine the clinical utility of multigene profiling assays (i.e., Oncotype DX, MammaPrint, Prosigna, EndoPredict, and Breast Cancer Index), not to identify which assay is better. No prospective studies have compared these assays head-to-head. Given that the assays use different scoring systems and classification systems, please refer to Table 3 for a summary of each of the assays. Further, this guideline does not cover the utility of multigene profiling assays in helping to guide clinical treatment decisions regarding the use of either neoadjuvant chemotherapy or radiation. Figure 1, Figure 2 and Figure 3 provide a summary of the recommendations in a decision tree.

### 4.1. Recommendation 1

In patients with early-stage estrogen receptor (ER)-positive/human epidermal growth factor 2 (HER2)-negative breast cancer, clinicians should consider using multigene profiling assays (i.e., Oncotype DX, MammaPrint, Prosigna, EndoPredict, and Breast Cancer Index) to help guide the use of systemic therapy.

#### 4.1.1. Qualifying Statement for Recommendation 1

- There is currently insufficient evidence to use multigene profiling assays among patients with either HER2-positive or triple-negative breast cancers;
- Multigene profiling assays are recommended for use in patients with lymph node-negative or lymph node-positive (1–3 lymph nodes) disease who are under consideration for adjuvant chemotherapy if the use is supported by other clinical, pathological, or patient-related factors. Clinical and pathological features include patient age, tumour grade, tumour size and nodal status;
- One multigene profiling assay should be requested per patient to guide a specific treatment decision. Requesting multiple tests with different multigene profiling assays on an individual tumour specimen to guide a single treatment decision is discouraged. Additional testing may be considered for patients with either repeat metachronous breast cancer diagnoses or synchronous breast cancer diagnoses where tumour specimens display varying morphologies, grade or hormone receptor status;
- Multigene profiling assays should be interpreted cautiously in pre-menopausal patients where a significant benefit from adjuvant chemotherapy may still exist despite a low-risk score.

#### 4.1.2. Key Evidence for Recommendation 1

Please see Key Evidence for Recommendations 2 through 4.

#### 4.1.3. Justification for Recommendation 1

The main purpose of most multigene profiling assays is to determine whether a tumour has a high or low risk for recurrence. The five multigene profiling assays considered in this guidance evaluate the intrinsic molecular characteristics of a tumour to prognosticate behaviour, with some being able to predict treatment benefit; however, the genes used to ascertain this predicted risk differ among assays. Although the results of different assays should be similar in terms of risk category, each individual assay uses a different scoring system, and the results may not be directly comparable. The value in multigene profiling is more evident and potentially limited to providing support for decision-making regarding systemic therapy when such decisions remain difficult for the clinician and patient, even after considering all clinical, pathologic, and patient-related factors. Although no males were included in any of the included studies, given the similarities in the management of male and female breast cancer, multigene profiling assays may be used in all individuals with early-stage ER-positive, HER2-negative invasive breast cancer.

Although multigene profiling assays may be used to guide treatment and ultimately improve patient outcomes, it is important to note the emotional impact such testing may have on patients, especially those who receive a high score. Clinician and patient discussions should be conducted concerning the implications of results.

### 4.2. Recommendation 2

In patients with early-stage node-negative ER-positive/HER2-negative disease, clinicians may use a low-risk result from Oncotype DX, MammaPrint, Prosigna, EndoPredict/EPclin, or Breast Cancer Index assays to support a decision not to use adjuvant chemotherapy.

#### 4.2.1. Qualifying Statement for Recommendation 2

- Patients < 50 years of age may still benefit from chemotherapy despite low-risk scores from multigene assay testing. Risk scores should be interpreted with caution, and decisions should be made while considering other clinical, pathological, or patient-related factors;
- Treatment decisions should be based on all available clinical and pathological information for each patient rather than depending entirely on multigene profiling test results;
- In patients with a low-grade tumour (i.e., grade 1) less than 1 cm in size, the Working Group members do not recommend a multigene assay profiling as this is unlikely to inform a treatment decision to use adjuvant chemotherapy. The Working Group would also not suggest the use of multigene profiling assays in patients who would not be willing or medically able to undergo chemotherapy.

#### 4.2.2. Key Evidence for Recommendation 2

For Oncotype DX, the evidence comes from one randomized controlled trial (RCT) [12,13] and two retrospective analyses of an RCT [14,15] with an overall low level of certainty as assessed using the GRADE approach.

- In the TAILORx trial [12], patients with a recurrence score (RS) ≤ 10 had an invasive disease-free survival (IDFS) rate of 94.0% and an overall survival (OS) rate of 98.0% with adjuvant endocrine therapy alone at five years and an IDFS rate of 84.0% and OS rate of 93.7% at nine years;
- No difference in freedom from distant recurrence (94.5% vs. 95.0%; *p* = 0.48), IDFS (83.3% vs. 84.3%; *p* = 0.26) or OS (93.9% vs. 93.8%; *p* = 0.89) was reported in patients with an RS of 11 to 25 between those who received endocrine therapy and chemoendocrine therapy at nine-year follow-up in the intent-to-treat population [12];
- In a subgroup analysis from the TAILORx trial among women aged ≤50 years [12], there was a significant benefit in those that received chemoendocrine therapy for IDFS with an RS of 16 to 20 (hazard ratio (HR), 1.90, 95% confidence interval (CI), 1.27 to 2.84; *p* = 0.0016) or 21 to 25 (HR, 1.70; 95% CI, 1.03 to 2.80; *p* = 0.035). This corresponded to a 1.6% reduction in the rate of distant recurrence among patients with an RS of 16 to 20 and a 6.5% reduction in the rate of distant recurrence among patients with an RS of 21 to 25 at nine-year follow-up;
- In an initial retrospective analysis of NSABP B20 [14], in patients with low (RS < 18) and intermediate scores (RS 18 to 20), there was no significant difference in 10-year freedom from distant recurrence between those that received chemotherapy and those that did not (95.6% vs. 96.8%; *p* = 0.61) and (89.1% vs. 90.9%; *p* = 0.39), respectively. There was a statistically significant interaction between chemotherapy treatment and RS score (*p* = 0.038). In the analysis by Geyer et al. [15], excluding patients with HER2-positive tumours, there was no benefit of chemotherapy in patients with low and intermediate scores. In a multivariable analysis, the test for interaction between chemotherapy and RS was statistically significant (*p* = 0.023) when controlling for patient age, tumour size, ER and progesterone receptor (PR) status, and tumour grade. Similarly, when the patients were recategorized by RS using TAILORx cut-offs, a statistically significant benefit was shown with the addition of chemotherapy for patients with an RS > 25, but there was no benefit in patients with RS < 11 and RS 11 to 25.

For MammaPrint, the evidence comes from one RCT [16,17] with a low level of certainty as assessed using the GRADE approach.

- In a prespecified exploratory subgroup analysis of the MINDACT trial of node-negative, ER-positive, HER2-negative patients, there was no significant difference in distant metastasis-free survival between patients who received chemotherapy and no chemotherapy in the high clinical risk and low genomic risk group (*p* = NR) or in the low clinical risk and high genomic risk group (*p* = NR). However, after a median follow-up of 8.7 years, there was a significant difference between the two treatment groups in the high clinical risk and low genomic risk group (HR, 0.60; 95% CI, 0.38 to 0.96; *p* = NR), but no significant difference in the low clinical risk and high genomic risk group (*p* = 0.815);
- In a predefined exploratory analysis of hormone receptor (HR)-positive, HER2-negative women at high clinical risk and low genomic risk, a significant chemotherapy benefit was shown (HR, 0.54; 95% CI, 0.30 to 0.98; *p* = NR) with an absolute difference of 5.0% in the rate of survival without distant metastases between the treatment groups in women 50 years of age or younger. No significant benefit was shown in women older than 50 years (HR, 0.82; 95% CI 0.55 to 1.24; *p* = NR). However, it is important to note that premenopausal patients were not mandated to receive ovarian suppression prior to treatment.

For Prosigna, the evidence comes from two predictive studies of retrospective analyses of RCTs [18,19] and three prognostic studies assessing late recurrence [20,21,22]. The prognostic studies did not maintain randomization from the original trials and, as a result, are treated as observational studies with very low certainty of the evidence as assessed using the GRADE approach.

- In both exploratory retrospective analyses of patients from the NCIC CTG MA.21 and DBCG 77B trials, the categorical Risk of Relapse (ROR) score was not predictive of response to chemotherapy regimen (*p* = 0.232) [18] for recurrence-free survival (RFS) or treatment (*p* = 0.10) for disease-free survival (DFS) [19], respectively;
- In a retrospective analysis of the ATAC trial, Sestak et al. [20] found that the risk of distant recurrence at 5 to 10 years was 1.4% (95% CI, 0.5 to 3.8) for low-risk patients.
- In a retrospective analysis of the ABSCG-8 trial, Filipits et al. [21] found the probability for 15-year distant RFS (DRFS) was 97.6% (95% CI, 94.7 to 98.9) for low-risk patients with a significant difference in late DRFS between patients in the high- vs. low-risk group (HR, 4.74; 95% CI, 1.89 to 11.87; *p* < 0.001);
- In a study combining both the ATAC trial and ABCSG-8 trial together [22], there was a significant difference in late distant recurrence (i.e., five to 10 years) between patients in the high- vs. low-risk group (HR, 5.49; 95% CI, 2.92 to 10.35; *p* = NR).

For EndoPredict, the evidence comes from two retrospective analyses of RCTs [20,23] assessing late recurrence. These prognostic studies did not maintain randomization from the original trials and, as a result. are treated as observational studies with very low certainty of the evidence as assessed using the GRADE approach.

- In a retrospective analysis of the ATAC trial, Sestak et al. [20] found the risk of distant recurrence for EPclin low-risk patients at 5 to 10 years was 4.3% (95% CI, 2.6 to 7.1);
- In an analysis of both the ABCSG-8 and ABCSG-6 trial together [23], there was a significant difference in DRFR from 5 to 15 years in women who were distant recurrence-free at 5 years between those with low and high EPclin scores (HR, 4.52; 95% CI, 2.65 to 7.72; *p* < 0.001).

For the Breast Cancer Index, the evidence comes from three retrospective analyses of RCTs [20,24,25] assessing late recurrence. These prognostic studies did not maintain randomization from the original trials and, as a result, are treated as observational studies with very low certainty of the evidence as assessed using the GRADE approach.

- In retrospective analyses of the ATAC trial [25], there was a significant difference between high Breast Cancer Index scores (BCI-high) and BCI-low groups (13.3% vs. 3.5%; HR, 2.97; 95% CI, 1.23 to 7.13; *p* = NR). In a multivariate analysis for late recurrence, the BCI molecular grade index MGI HOXB13/IL17BR (MGI H/I) was prognostic for risk of distant late recurrence in node-negative (HR, 1.95; 95% CI, 1.22 to 3.14) and node-negative HER2-negative populations (HR, 2.12; 95% CI, 1.30 to 3.47). Sestak et al. [20] found the risk of distant recurrence at 5 to 10 years was 2.6% (95% CI, 1.3 to 5.0) for low-risk patients and 15.9% (95% CI, 8.9 to 27.6) for high-risk patients;
- Zhang et al. [24] found there was a significant difference in late DRFS between the BCI-low, BCI-intermediate, and BCI-high-risk groups for patients in both the Stockholm cohort and the multi-institutional cohort (*p* = 0.0152 and *p* = 0.0002, respectively). In a multivariate Cox regression including clinicopathologic variables, BCI was significant for ER-positive, HER2-negative patients in both the Stockholm cohort (HR, 3.50; 95% CI, 1.09 to 11.21; *p* = 0.035) and the multi-institutional cohort (HR, 9.24; 95% CI, 2.85 to 30.0; *p* = 0.0002).

#### 4.2.3. Justification for Recommendation 2

Patients from the Consultation Group rated both recurrence risk and survival as critical outcomes, along with quality of life and adverse events. The benefits of withholding adjuvant chemotherapy would be large and acceptable to patients when there are no significant differences in survival benefits. Prognostic studies from Prosigna and EndoPredict demonstrate a low risk of late recurrence, which would make it acceptable to withhold chemotherapy given the potential side effects and toxicity associated with adjuvant chemotherapy. The Working Group notes that although the overall certainty of the evidence is low for both Oncotype DX and MammaPrint, the TAILORx and MINDACT trials provide the strongest available evidence and best trial design available for this population. Given the similarities in the management of male and female breast cancer, these data can be generalized to all individuals with early-stage ER-positive, HER2-negative invasive breast cancer.

### 4.3. Recommendation 3

In patients with node-negative ER-positive/HER2-negative disease, clinicians may use a high-risk result from Oncotype DX to support a decision to offer chemotherapy. A high Oncotype DX recurrence score is capable of predicting adjuvant chemotherapy benefit.

#### 4.3.1. Qualifying Statement for Recommendation 3

MammaPrint, Prosigna, EndoPredict or EPclin and the Breast Cancer Index do not have sufficient evidence to support a predictive benefit of adjuvant chemotherapy among clinically low-risk breast cancer patients whose multigene profiling testing indicates a high-risk score.

#### 4.3.2. Key Evidence for Recommendation 3

The evidence comes from one RCT [12,13,26] and two retrospective analyses of an RCT [14,15] with an overall low level of certainty as assessed using the GRADE approach.

- In the TAILORx trial [26], the rate of freedom from recurrence of breast cancer at a distant site for high-risk patients (RS 26–100) treated with endocrine therapy plus adjuvant chemotherapy was 93% at five years and 86.8% at nine years;
- In a retrospective analysis of the NSABP B20 trial [14], patients with high RS (RS ≥ 31) experienced a large chemotherapy benefit (60.5% vs. 88.1%; relative risk (RR), 0.26; 95% CI, 0.13 to 0.53) and a statistically significant interaction between chemotherapy treatment and RS score (*p* = 0.038). In the second re-analysis by Geyer et al. [15], a benefit of chemotherapy remained for patients with high RS (HR 0.18; 95% CI, 0.07 to 0.47; *p* < 0.001); however, there was no benefit of chemotherapy in patients with RS < 18 and RS 18 to 30. In a multivariable analysis, the test for interaction between chemotherapy and RS was statistically significant (*p* = 0.023) when controlling for patient age, tumour size, ER and PR status, and tumour grade. Similarly, when the patients were recategorized by RS using TAILORx cut-offs, a statistically significant benefit was shown with the addition of chemotherapy for patients with an RS > 25. In a multivariable analysis, the test for interaction between chemotherapy and RS was statistically significant (*p* = 0.014) when controlling for patient age, tumour size, ER and PR status, and tumour grade. It is important to note that the patients included in the tamoxifen-only arm were used in the initial development of the Oncotype DX assay, and as a result, these results may be confounded.

#### 4.3.3. Justification for Recommendation 3

Patients from the Consultation Group rated both recurrence rate and invasive DFS as critical outcomes along with quality of life and adverse events. The Working Group determined that the beneficial effects of lower recurrence rates and higher survival rates outweigh the adverse effects of adjuvant chemotherapy.

Given the similarities in the management of male and female breast cancer, these data can be generalized to all individuals with early-stage ER-positive, HER2-negative invasive breast cancer.

### 4.4. Recommendation 4

In postmenopausal patients with ER-positive/HER2-negative tumours and one to three nodes involved (N1a disease), clinicians may withhold chemotherapy based on a low-risk Oncotype DX or MammaPrint score if the decision is supported by other clinical, pathological, or patient-related factors.

#### 4.4.1. Qualifying Statement for Recommendation 4

- Premenopausal patients < 50 years of age have a significant benefit from chemotherapy despite low-risk scores from multigene assay testing. Risk scores should be interpreted with caution, and decisions should be made while considering other clinical, pathological, or patient-related factors;
- It is uncertain whether at least some of the benefit of chemotherapy among premenopausal patients may be due to chemotherapy-induced amenorrhea versus the cytotoxic effects of treatment;
- The Prosigna, EndoPredict/EPclin, and Breast Cancer Index assays are capable of identifying low-risk node-positive patients whose prognostic outcomes are favorable; however, these assays have not demonstrated predictive evidence to support withholding adjuvant chemotherapy among higher risk, node-positive, ER-positive, HER2-negative breast cancer patients.

#### 4.4.2. Key Evidence for Recommendation 4

For Oncotype DX, the evidence comes from one RCT [27], the RxPONDER trial, and a retrospective study of the SWOG 8814 trial [28] with low certainty of the evidence as assessed using the GRADE approach.

- The RxPONDER trial [27] reported there was no significant difference in IDFS at five years between patients (RS ≤ 25) who received chemoendocrine therapy or endocrine therapy (92.2% vs. 91.0%; HR, 0.86; 95% CI, 0.72 to 1.03; *p* = 0.10). The interaction between chemotherapy benefit and continuous recurrence score was not statistically significant for IDFS when controlling for continuous RS, menopausal status, and treatment group (*p* = 0.35);
  ○ In a prespecified analysis, a significant interaction was found between the addition of adjuvant chemotherapy and menopausal status (*p* = 0.008), allowing for subgroup analysis by menopausal status. In postmenopausal women, there was no significant difference in IDFS between those who received chemoendocrine therapy or endocrine therapy (91.3% vs. 91.9%; HR, 1.02; 95% CI, 0.82 to 1.26; *p* = 0.89);
  ○ In premenopausal women, a significant benefit was found in IDFS for women who received chemoendocrine therapy (93.9% vs. 89.0%; HR, 0.60; 95% CI, 0.43 to 0.83; *p* = 0.002). In premenopausal women who were 50 years old or older, there was no significant chemotherapy benefit (HR, 0.98; 95% CI, 0.54 to 1.78); however, in premenopausal women younger than 50 years old, a significant chemotherapy benefit was observed (HR, 0.48; 95% CI, 0.32 to 0.72; *p* = NR). The interaction between age and chemotherapy benefit in premenopausal women was not significant (*p* = 0.06);
- In a retrospective analysis of the SWOG-8814 trial [28], there was no significant benefit for DFS or OS between patients who received either tamoxifen alone or cyclophosphamide, doxorubicin, and 5-fluorouracil (CAF) followed by tamoxifen at 10 years for those with RS < 18 (*p* = 0.97 and *p* = 0.68, respectively) or RS between 18 and 30 (*p* = 0.48 and *p* = 065, respectively). For DFS, there was no significant interaction between RS and treatment (*p* = 0.053); however, when assessing the first five years, a significant interaction was seen between RS and treatment for both DFS and OS (*p* = 0.029 and *p* = 0.016, respectively) but not after five years (*p* = 0.58 and *p* = 0.87, respectively).

For MammaPrint, the evidence comes from one RCT [16,17] with a low level of certainty as assessed using the GRADE approach.

• In node-positive patients in the MINDACT trial, there was no significant difference between patients who received chemotherapy and no chemotherapy in the high clinical risk and low genomic risk group for distant metastasis-free survival after a median follow-up of five years (absolute benefit of 0.7% in the chemotherapy arm) [16]; (*p* = 0.724) or eight years (absolute benefit of 1.3% in the chemotherapy arm; *p* = NS) [17]. The number of node-positive patients in the low clinical risk and high genomic risk was too small to be analyzed.

#### 4.4.3. Justification for Recommendation 4

Patients from the Consultation Group rated both recurrence risk and survival as critical outcomes, along with quality of life and adverse events. The benefits from withholding adjuvant chemotherapy would be large and acceptable to patients when there are no significant differences in survival benefits. Although favourable prognostic data exist for late recurrence with Prosigna, EndoPredict and the Breast Cancer Index, given the increased clinical risk in lymph node-positive patients, strong predictive data regarding the use of these assays are needed. Given the similarities in the management of male and female breast cancer, these data can be generalized to all individuals with early-stage ER-positive, HER2-negative invasive breast cancer.

### 4.5. Recommendation 5

The evidence to support the use of molecular profiling to select the duration of endocrine therapy is evolving. In patients with ER-positive disease, clinicians may consider using a Breast Cancer Index (H/I) high assay result to support a decision to extend adjuvant endocrine therapy if the decision is supported by other clinical, pathological, or patient-related factors.

#### 4.5.1. Qualifying Statement for Recommendation 5

- While a number of studies have demonstrated the clinical utility of BCI for extending adjuvant endocrine therapy, the preliminary results of the NSABP B42 trial are negative, leading to some uncertainty. Treatment decisions should be based on all available clinical and pathological information for each patient rather than depending only on multigene profiling tests;
- MammaPrint, Oncotype DX, Prosigna, and EndoPredict currently have insufficient evidence to guide the extension of adjuvant endocrine therapy; however, these molecular assays may prognosticate a very low rate of disease recurrence that might not justify an extension of endocrine therapy.

#### 4.5.2. Key Evidence for Recommendation 5

For the Breast Cancer Index, the evidence comes from four retrospective analyses of RCTs [29,30,31,32]. One [32] is currently available in abstract form, with low certainty of evidence as assessed using the GRADE approach.

- In a retrospective review of the NSABP B42 trial [32], currently in abstract form, there was no significant difference between receiving an additional five years of letrozole or placebo for recurrence-free interval in those who were BCI (H/I)-low (HR, 0.69; 95% CI, 0.43 to 1.11; *p* = 0.13) or BCI (H/I)-high (HR, 0.83; 95% CI, 0.55 to 1.26; *p* = 0.38). There was no significant interaction between BCI (H/I) level and treatment (*p* = 0.55) for recurrence-free interval, breast cancer-free interval (*p* = 0.07), DFS (*p* = 0.62), or distant recurrence (*p* = 0.14);
- In the translation IDEAL study [31], there was significant benefit in risk of recurrence for BCI (H/I)-high patients who received five years of extended letrozole (HR, 0.42; 95% CI, 0.21 to 0.84; *p* = 0.011) with an absolute reduction of recurrence risk of 9.8%; however, this benefit was not observed in BCI (H/I)-low patients (HR, 0.95; 95% CI, 0.58 to 1.56; *p* = 0.835). Similarly, in patients treated with primary adjuvant endocrine therapy with an AI, BCI (H/I)-high patients received a significant benefit from extended letrozole (HR, 0.34; 95% CI, 0.16 to 0.73; *p* = 0.004), while no benefit was seen in BCI (H/I)-low patients (HR, 0.90; 95% CI, 0.53 to 1.55; *p* = 0.712). There was a significant interaction between BCI (H/I) level and treatment in both the overall population (*p* = 0.045) and in the subgroup of patients who received primary adjuvant endocrine therapy with an AI (*p* = 0.025) after adjusting for age, tumour grade, pT stage, pN stage, prior endocrine therapy and prior chemotherapy;
- In the Trans-aTTom study [30], consisting of node-positive patients only, those classified as BCI (H/I)-high showed a significant benefit from extended tamoxifen (HR, 0.35; 95% CI, 0.15 to 0.86; *p* = 0.027) with an absolute recurrence risk difference of 10.2%. There was a significant interaction between continuous BCI (H/I) and extended tamoxifen treatment (*p* = 0.012) after adjusting for age, tumour size, tumour grade, and ER and PR status;
- In the retrospective review of the NCIC CTG MA 17 trial [29], for patients with high H/I, there was a significant difference in the five-year RFS of 73% (95% CI, 56.6 to 84.1) and 89.5% (95% CI, 80.3 to 94.5) for patients receiving placebo and letrozole, respectively, with an absolute risk of reduction of 16.5% (*p* = 0.007). In an adjusted model, high H/I was significantly associated with patient benefit from letrozole (odds ratio (OR), 0.32; 95% CI, 0.14 to 0.72; *p* = 0.006). The interaction between H/I and letrozole therapy was significant (*p* = 0.03).

#### 4.5.3. Justification for Recommendation 5

Patients from the Consultation Group rated both the recurrence risk and RFS as critical outcomes along with quality of life and adverse events. The Working Group determined the beneficial effects of lower recurrence and higher survival rates outweigh the adverse effects of extended adjuvant endocrine therapy. This recommendation can be generalized to all patients with node-negative and -positive, ER-positive breast cancer.

The Working Group acknowledges the emerging evidence for MammaPrint in this area [33] as well as the retrospective study of the NSABP B42 trial for BCI [32]; however, abstracts of studies are insufficient to make recommendations. Translational studies from the IDEAL, Trans-aTTom, and NCI CCTG MA 17 clinical trials all demonstrated a clinical benefit from extended adjuvant endocrine therapy among patients with a Breast Cancer Index (H/I) high assay result; however, the results from the recent analysis of the NSABP B42 trial were negative. While the NSABP B42 trial is only presented as an abstract, this preliminary result does raise some uncertainty regarding the predictive capacity of BCI, and the Working Group has thus issued a weak recommendation for BCI (H/I) testing to guide extended adjuvant endocrine therapy.

## 5. Conclusions

The use of multigene profiling assays for early-stage, node-negative, ER-positive, HER2-negative breast cancer is well established. A variety of assays can be used to identify low-risk patients with a favourable disease prognosis who can be safely treated with endocrine therapy alone. We have now updated our clinical practice guideline demonstrating that both Oncotype DX and MammaPrint can also be used in patients with limited lymph node-positive disease (pN1a or 1–3 positive lymph nodes) to help identify patients at low risk who do not require treatment with adjuvant chemotherapy. Caution should be used in interpreting low-risk multigene assay scores in premenopausal women where a small adjuvant chemotherapy benefit exists even among patients with low-risk scores. The role of multigene profiling assays to guide clinical decisions regarding the duration of adjuvant endocrine therapy is also emerging, and the Breast Cancer Index may be considered for use in aiding decisions regarding extending adjuvant endocrine therapy. Further research is required, and future studies will also help clarify the potential use of multigene profiling assays to guide clinical decision making regarding neoadjuvant chemotherapy and adjuvant radiation therapy. Overall, multigene profiling assays are valuable clinical tools to be discussed with patients to help guide and facilitate personalized clinical treatment decisions for adjuvant systemic therapy in patients with early-stage breast cancer.

## Figures and Tables

**Table 1:** Responses to nine items on the targeted peer reviewer questionnaire.

Question	Reviewer Ratings (*n =* 1)				
	Lowest Quality				Highest Quality
	(1)	(2)	(3)	(4)	(5)
Rate the guideline development methods.	0	0	0	0	1
Rate the guideline presentation.	0	0	0	0	1
Rate the guideline recommendations.	0	0	0	1	0
Rate the completeness of reporting.	0	0	0	0	1
Does this document provide sufficient information to inform your decisions? If not, what areas are missing?	0	0	0	0	1
Rate the overall quality of the guideline report.	0	0	0	0	1
	Strongly disagree		Neutral		Strongly agree
	(1)	(2)	(3)	(4)	(5)
I would make use of this guideline in my professional decisions.	0	0	0	0	1
I would recommend this guideline for use in practice.	0	0	0	0	1
What are the barriers or enablers to the implementation of this guideline report? None were noted.					

**Table 2:** Responses to four items on the professional consultation survey.

General Questions	Overall Guideline Assessment (*n* = 25)				
	Lowest Quality				Highest Quality
	(1)	(2)	(3)	(4)	(5)
Rate the overall quality of the guideline report.	0	0	1	14	10
	Strongly disagree				Strongly agree
	(1)	(2)	(3)	(4)	(5)
I would make use of this guideline in my professional decisions.	0	0	4	9	12
I would recommend this guideline for use in practice.	0	0	2	9	14
What are the barriers or enablers to the implementation of this guideline report? Availability, accessibility, and funding of assays Limitations in access to multidisciplinary care in remote areas Education Bureaucracy in filling out online forms for Ministry approval					

**Table 3:** Summary of assay characteristics.

Characteristics/Assay Name	Oncotype DX	Prosigna	MammaPrint	EndoPredict	Breast Cancer Index
Tissue Required	FFPE	FFPE	FFPE or fresh tissue	FFPE	FFPE
Technique	qRT-PCR	qRT-PCR and nCounter DX Analysis System	Microarray	qRT-PCR	qRT-PCR
Assay Output	RS (0–100)	Intrinsic subtype and ROR score (0–100)	MammaPrint Index Risk of distant recurrence at 5 years	EPclin score (1–6) Molecular score (1–15)	BCI score (0–10) and BCI (H/I) low and BCI (H/I) high (ratio HoxB13 and interleukin-17B receptor)
Categories for Risk Measurement	TAILORx categories Low: ≤15 Intermediate: 16–25 High: 26–100 Pre-TAILORx categories Low: <18 Intermediate: 18–30 High: ≥31	LN-negative Low: 0–40 Intermediate: 41–60 High: 61–100 LN-positive (1–3 nodes) Low: 0–40 High: 41–100	Low: 0 to 1 High: −1 to 0	EPclin score Low: < 3.3 High: ≥ 3.3 Molecular score Low: < 5 High: ≥ 5	BCI predictive H/I Low: <0.06 High: ≥0.06 BCI prognostic node-negative Low: <5.0825 Intermediate: 5.0825–6.5025 High: ≥6.5025 BCI prognostic node-positive Low: <6.93 High: ≥6.93
Regulatory Approval or Endorsement	Assay conducted in centralized Exact Science’s CLIA-certified lab	FDA cleared for decentralized testing (2014)	FDA cleared for Agendia centralized lab testing in FFPE (2015)	CE Mark for decentralized testing (2012)	Assay conducted in centralized CAP/CLIA-certified lab
Manufacturer	Exact Sciences Corp.	Veracyte	Agendia	Myriad Genetics, Inc.	Biotheranostics, Inc.
Testing Location	Central (1 laboratory in US)	Various labs across US, UK	Central (1 laboratory in the Netherlands, 1 in US)	Central laboratory in the US	Central (1 laboratory in US)
Genes, *n*	21-gene assay	50-gene assay	70-gene assay	12-gene assay EPclin score: 12-gene assay plus tumour size and nodal status	*HOXB13:IL17BR* expression ratio (H/I) and Molecular Grade Index

Abbreviations: BCI (H/I), Breast Cancer Index (HOXB13/IL17BR); CAP: College of American Pathologists; CLIA: Clinical Laboratory Improvement Amendments; EPclin, EndoPredict clinical score; ER, estrogen receptor; FDA, Food and Drug Administration; FDA: Food and Drug Administration; FFPE, formalin-fixed paraffin-embedded; LN, lymph node; qRT-PCR, quantitative reverse-transcription polymerase chain reaction; ROR: risk of recurrence; RS, recurrence score; UK: United Kingdom; US, United States.
